# Christopher Cowell, *Form Follows Fever: Malaria and the Construction of Hong Kong, 1841-1849*

**DOI:** 10.1093/shm/hkaf019

**Published:** 2025-03-03

**Authors:** Ria Sinha

**Affiliations:** The University of Hong Kong, Hong Kong SAR

Describing the newly acquired Hong Kong as a ‘pest-house’, British Vice Consul Henry Charles Sirr was unequivocal in his views about the island’s insalubrity and inhospitable environment.^1^ Plagued by disease and lacking permanent infrastructure, incoming officials, troops, traders, civilians and labourers faced harsh realities and a terrifying mortality rate. Malaria, the quintessential ‘tropical disease’ that had long impeded European empire building, was a major cause of death, incapacitating entire military regiments who were inadequately housed in poor conditions. Yet as its author is at pains to point out, *Form Follows Fever* is not a malaria history *per se*.

Christopher Cowell intuitively draws on his interdisciplinary expertise at the intersection of architectural and medical history, and a rich array of textual primary sources and visual renderings, to present an original and unusually detailed analysis of Hong Kong’s difficult first colonial decade. The island territory was a blank slate in some respects; the early years were about fashioning a habitable and functional city, but there were inherent physical and microbial limitations as to how much the island could be modified, which exacerbated local and transnational colonial politics. Attempts to transform the island into a trade hub, a military command and colonial outpost were marked by successes and failures, and Cowell contends that the mixed econopolitical interests of different stakeholders complicated the picture. The narrative picks up on these tensions, yet it also interrogates suppositions that Hong Kong might have been abandoned by the British on health grounds for the more temperate climes of Chusan to the north, but the island was strategically too important, even if it cost lives.

Early construction choices were often ill-informed, based on aesthetics rather than suitability. Location too was paramount but ‘picturesque’ settings were also some of the unhealthiest on the island. As the ‘form’ of the city, its government buildings, residences, military posts, hospitals, roads and other public amenities, emerged and evolved mistakes were made, ‘redrawn’ and ‘remade’ through trial and error. The malarial lens employed by Cowell is one that serves its purpose well, showing how an enigmatic disease that was imagined as embedded into the very fabric of the island altered urban planning and designated certain areas as ‘unlivable’. Unlike the sanitary debates that would take place in the latter half of the nineteenth century, malaria was not linked to the indigenous population, but to the environment. The ill-fated experiences of other colonial settlements, including India and Southeast Asia were referenced and drawn on to overcome the challenges of inhabiting and surviving tropical climes. Indeed, the remnants of Hong Kong’s early architectural form, low-rise ‘bungalow’ style buildings with shaded open verandahs facing into the cleansing breezes, are still evident, albeit disappearing year-on-year.

Organised largely chronologically, the book expands on two major malaria epidemics that plagued Hong Kong in 1843 and 1848 killing a significant proportion of the mixed population, to demonstrate how the endemic and epidemic potential of the disease materially influenced and modified the siting of residential, military and business districts. The beautiful cartographic illustrations that intersperse the text demonstrate how mapping became a powerful tool in creating medical topographies (not just in Hong Kong), to delineate healthy and diseased environs. Cowell’s generous but judicious use of different views and renderings of Hong Kong, two-dimensional and three-dimensional, from above and from the ground, macro- and microscalar, allows the reader to engage with the problems and expectations on the new colony, from the home (British) government, the military, traders and the mixed civilian population.

The book’s focus on the first decade is intentional and should not be seen as too narrow or delimiting. Nonetheless, readers are hopefully not left with the impression that malaria and its entanglement with place and Hong Kong’s built environment was a short-term issue, conquered by superior colonial knowledge and practices. Conversely, the disease would persist for another 150 years, vexing urban planners and their infrastructure projects well into the twentieth century, continuing to shape where and how people lived and worked. Cowell does acknowledge that despite his ‘forensic approach’ there is space to expand on this study, to comprehend the lives and deaths of early residents caught in the socioecological web of an inexplicable disease and colonial ambitions. In summary, *Form Follows Fever* is an exceptional contribution to scholarship on Hong Kong’s early history, offering a multilayered and graphic illustration of early design and construction challenges, set against the irrepressible backdrop of endemic malaria. The book will appeal to a niche but diverse subset of academics, professionals and students, including architects, historians of medicine and empire, as well as anyone interested in Hong Kong history.

## Footnotes

1 H. C. Sirr. *China and the Chinese: Their Religion, Customs, Character and Manufactures: The Evils Arising from the Opium Trade: With a Glance at Our Religious, Moral, Political and Commercial Intercourse with the Country* (London, England: W.S. Orr, 1849), 17.

